# The defense and signaling role of NADPH oxidases in eukaryotic cells: Review

**DOI:** 10.1007/s10354-018-0640-4

**Published:** 2018-08-06

**Authors:** Michael Breitenbach, Mark Rinnerthaler, Manuela Weber, Hannelore Breitenbach-Koller, Thomas Karl, Paul Cullen, Sukaniya Basu, Dana Haskova, Jiri Hasek

**Affiliations:** 1 Department of Bioscienes, University of Salzburg, Salzburg, Austria; 2 Department of Biological Sciences, University at Buffalo, The State University of New York, Buffalo, USA; 3 Laboratory of Cell Reproduction, Institute of Microbiology of AS CR, v.v.i., Prague, Czech Republic

**Keywords:** Chronic granulomatous disease, *Saccharomyces cerevisiae*, Actin cytoskeleton, Nox enzymes, Reactive oxygen species, Septische Granulomatose, *Saccharomyces cerevisiae*, Aktinzytoskelett, Nox-Enzyme, Reaktive Sauerstoffspezies

## Abstract

This short review article summarizes what is known clinically and biochemically about the seven human NADPH oxidases. Emphasis is put on the connection between mutations in the catalytic and regulatory subunits of Nox2, the phagocyte defense enzyme, with syndromes like chronic granulomatous disease, as well as a number of chronic inflammatory diseases. These arise paradoxically from a lack of reactive oxygen species production needed as second messengers for immune regulation. Both Nox2 and the six other human NADPH oxidases display signaling functions in addition to the functions of these enzymes in specialized biochemical reactions, for instance, synthesis of the hormone thyroxine. NADPH oxidases are also needed by *Saccharomyces cerevisiae* cells for the regulation of the actin cytoskeleton in times of stress or developmental changes, such as pseudohyphae formation. The article shows that in certain cancer cells Nox4 is also involved in the re-structuring of the actin cytoskeleton, which is required for cell mobility and therefore for metastasis.

## Zusammenfassung

In diesem kurzen Übersichtsbeitrag fassen wir den klinischen und biochemischen Wissensstand zu den sieben menschlichen NADPH-Oxidasen zusammen. Ein Schwerpunkt liegt dabei auf dem Zusammenhang zwischen Mutationen in den katalytischen und regulatorischen Untereinheiten von Nox2 – der NADPH-Oxidase der Phagozyten mit Abwehrfunktion – und Syndromen wie der septischen Granulomatose, aber auch einer Reihe von chronisch-entzündlichen Erkrankungen. Hervorgerufen werden diese durch einen Mangel an reaktiven Sauerstoffspezies, welche als Produkte der NADPH-Oxidase-Reaktion für die Immunregulation notwendig sind. Sowohl Nox2 als auch die anderen sechs menschlichen NADPH-Oxidasen haben neben ihren Funktionen in spezialisierten biochemischen Reaktionen (Beispiel: Thyroxinsynthese) auch eine Funktion in der Signaltransduktion. In der Hefe *Saccharomyces cerevisiae* wird die NADPH-Oxidase Yno1 als Signalgeber für die Restrukturierung des Aktinzytoskeletts gebraucht, z. B. unter Stress oder bei der Bildung von Pseudohyphen. In bestimmten Tumorzellen ist die NADPH-Oxidase Nox4 ebenfalls an der Restrukturierung des Aktinzytoskeletts beteiligt – eine Voraussetzung für die Zellmobilität und damit auch für die Metastasierung dieser Tumoren.

## Introduction

This review paper presents: (i) an overview of the present state of knowledge of the human NADPH oxidases and their clinical relevance; (ii) the authors’ own published analysis of the properties and biological significance of the only NADPH oxidase of *Saccharomyces cerevisiae*, Yno1; and (iii) the function of both Yno1 and Nox4 in the regulation of the actin cytoskeleton, which may be important for the mobility of cancer cells, as studied in a neuroblastoma cell line.

The production of superoxide and a host of radical and non-radical molecular species derived thereof, collectively called reactive oxygen species (ROS) and through reaction with nitric oxide (NO), reactive nitrogen species (RNS), serves two functions: defense against invading organisms as part of the innate immune system and signaling. There is accumulating evidence for an involvement in an increasing number of biologically important signal transduction processes. NADPH oxidases are not the only intracellular sources of ROS. Normal cellular metabolism produces hydrogen peroxide (H_2_O_2_) (examples: peroxisomal oxidases that convert O_2_ to H_2_O_2_; the cytochrome P450 system [1]; xanthine oxidase reaction and certain stressful or pathological conditions produce superoxide, and indirectly H_2_O_2_ [examples: aged mitochondria through “leakage” of single electrons in complexes I and III [2]; production of methemoglobin in certain mutant hemoglobins like HbS [3]]. Additionally, deleterious ROS are also produced by biogenic and non-biogenic environmental attacks from outside [for instance as a secondary consequence of ionizing radiation, IR], leading to oxidative stress).

One of the most prominent authors in the field of oxidative stress [4, 5] discriminates between oxidative eustress (broadly equivalent to ROS signaling) and oxidative distress, which is broadly equivalent to a much larger level of ROS, ultimately leading in many cases to a series of events that can culminate in cell death. It may be considered self-explanatory that signaling (nearly exclusively through hydrogen peroxide) requires much lower levels of ROS and only a transient elevation in a highly localized fashion in the cell, while high local concentrations can be reached in certain cellar compartments in antimicrobial defense. In both cases, degradation of ROS is of utmost importance after the job is done, otherwise the persistence of ROS would lead to chronic inflammatory diseases. Examples of these are discussed below. The first and best known example of ROS signaling is regulation of the activity of protein phosphotyrosine phosphatases (PTPs) [6] in the course of growth factor stimulation of cultured human cells [7]. Activation of target proteins by phosphorylation is increased when the counteracting protein phosphatase PTP is reversibly inhibited by the formation of sulfenic acid in critical cysteine groups.

The larger gene and protein family discussed here comprises not only Nox enzymes but also ferric reductases, which play an important role in the mechanism and regulation of iron uptake into cells. The whole superfamily is therefore called IMR (integral membrane reductase) protein superfamily. The previously held opinion that only multicellular eukaryotic species express Nox enzymes has been disproved by our analysis of the *S. cerevisiae* Nox enzme, Yno1 [8], and by the recent finding and functional analysis of bacterial Nox enzymes [9].

The most important authoritative and comprehensive review article dealing with the human NADPH oxidases (Nox enzymes, for short), was published 10 years ago [10]. The most important conclusions of these authors, together with the relevant literature that has appeared in the meantime, are summarized below in the first part of this article.

### Structure and reaction scheme of NADPH oxidases (Nox enzymes)

**Fig. 1:**
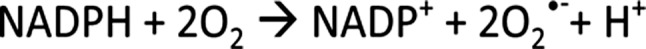
Reaction equation of NADPH oxidases. The reaction takes place in a vectorial manner. On the cytoplasmic side, NADPH and O_2_ enter the reaction and electron pairs are first transferred from NADPH to FADH. In the membrane, single electrons are transferred to the first and then to the second b‑type cytochrome, and on the opposite side of the membrane, single electrons are transferred to the O_2_ molecule, producing two molecules of superoxide in the lumen of the endoplasmic reticulum (ER). Further metabolic reactions are not completely known, leading to the probable signaling substance, hydrogen peroxide. This could be through the NADPH oxidase itself (as shown for human Nox4) or through a tightly coupled superoxide dismutase enzyme

**Fig. 2:**
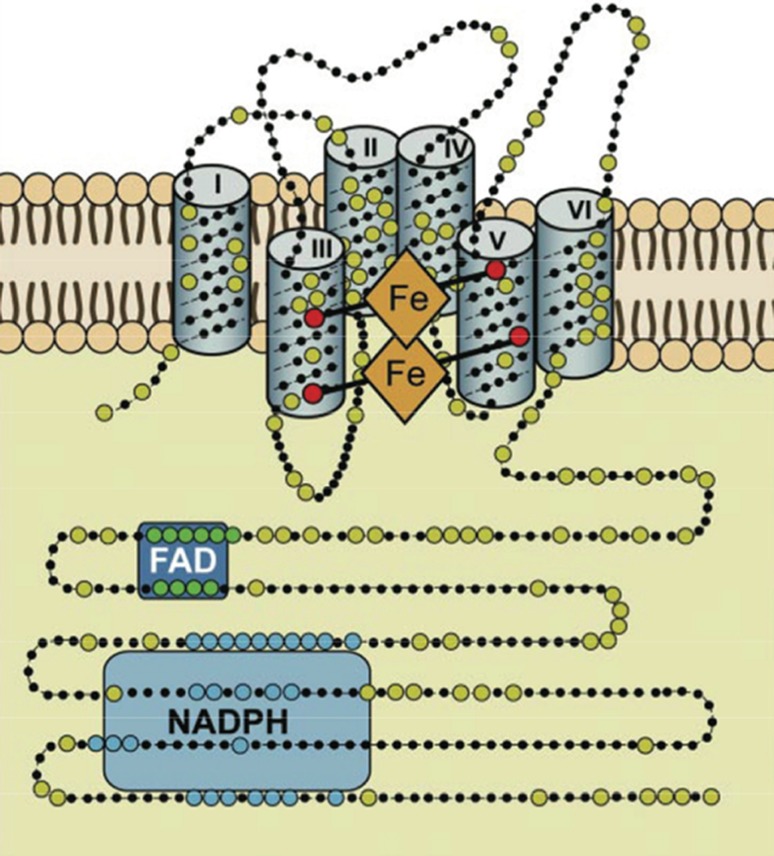
General scheme of NADPH oxidases in membranes. This structural model is based on bioinformatics, cell fractionation, and biochemical data concerning the human Nox enzymes (NOX1, 2, 3, and 4), but is also correct for other Nox enzymes. Nox enzymes typically comprise around 500 amino acids and are exclusively located in lipid bilayer membranes, like the plasma membrane or the endoplasmic reticulum membrane. *Large dots* are highly conserved amino acids. The reaction center transferring a single electron to oxygen is the upper b‑type heme in this scheme. The enzyme consists of six transmembrane helices. The two b‑type hemes are coordinated with histidine residues between helices III and V. The enzyme contains binding sequences for NADPH as well as for flavin adenine dinucleotide (*FAD*) in its cytoplasmic tail. (Modified from [10])

**Fig. 3:**
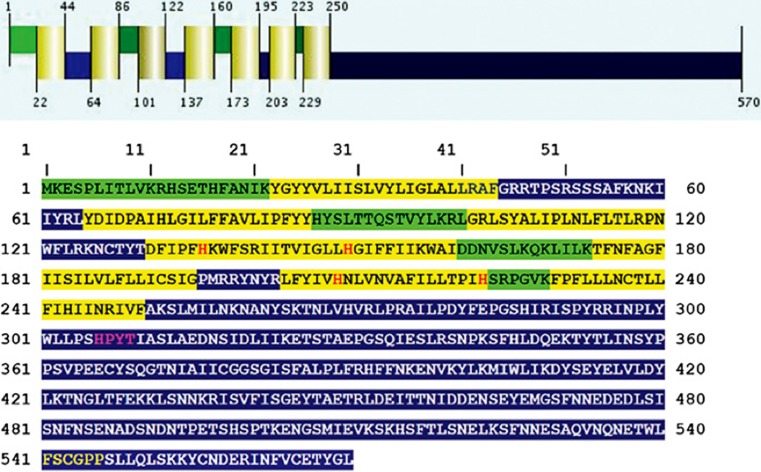
Bioinformatic structure predictions for the yeast NADPH oxidase Yno1. The upper part of the figure, the secondary structure and the transmembrane helices (*highlighted in yellow*). The luminal regions, which reside presumably in the lumen of the endoplasmic reticulum, are *highlighted in green*. The cytoplasmic regions are *highlighted in blue*. The four conserved histidine residues responsible for coordinating the two heme groups are *marked with* *red letters*. The NADPH binding site is *marked with yellow letters* and the flavin adenine dinucleotide (FAD) binding site is *highlighted with purple letters.* (Modified from [8])

All known Nox enzymes are transmembrane proteins which, in a vectorial way, catalyze the one-electron reduction of dioxygen (O_2_) to produce superoxide (O_2_^−^), an anion radical (see Fig. 1). The one-electron transmembrane reduction reaction is accomplished by four different sequential redox-active co-factors: NADPH, FADH, and two non-identical b‑type cytochromes. Fig. 2 shows the binding consensus sequences for these co-factors. As a negative charge is created on the outside of the membrane, this must be compensated by a proton that is transported through the membrane. No high-resolution three-dimensional structure of a whole-length Nox enzyme is known, mainly due to the reluctance of membrane proteins to yield crystals suitable for X‑ray diffraction. However, in 2017 the dehydrogenase domain and the transmembrane domain of the cyanobacterial Nox5 ortholog were resolved to 2.2 and 2.05 A, respectively [11]. The hypothetical structure shown in Fig. 2 is in excellent agreement with the combined structures of the two domains. This structure can beautifully explain the vectorial transfer of single electrons to form the superoxide radical anion. Additionally, the now available crystallographic structure confirms previous biochemical experiments in vivo and in vitro, including in vitro mutagenesis studies. Bioinformatic analysis identifies transmembrane helices, binding histidines for the two cytochromes b, consensus binding sequences for the co-factors NADPH and FADH and show which parts of the enzyme are located in the inside (cytoplasm in the case of Nox2) and on the outside of the membrane (extracellular space) (Figs. 2 and 3).

### The human Nox enzymes

**Fig. 4:**
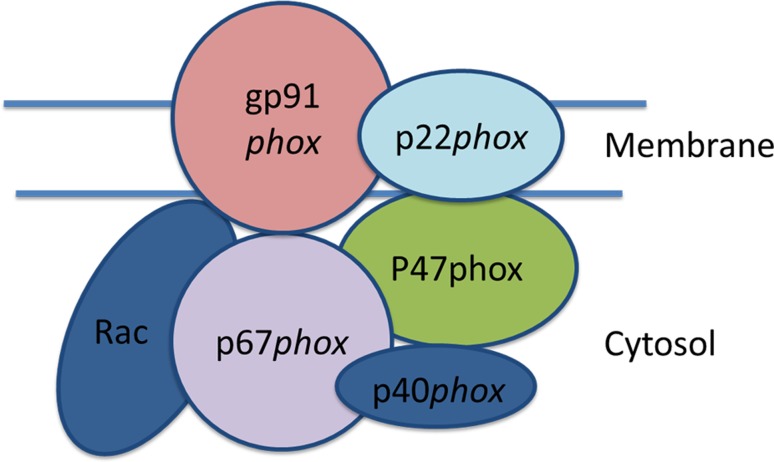
The disposition of Nox2 in its active state at the plasma (phagosome) membrane. The intrinsic membrane protein, p22, is associated with the catalytic subunit, gp91. The other subunits are only transiently associated with gp91 during the phase of the oxidative burst and are soluble cytoplasmic proteins. (Modified from [13])

Based on sequence similarity and biochemical activity, seven genes in the human genome have been annotated as being NADPH oxidases [10]. Before a discussion of some salient features of these seven enzymes is presented, the reader’s attention should be brought to the fact that research performed over the last 10 years has increasingly shown that organ- and cell-specific expression, as well as biochemical activity of these enzymes is not as highly specialized and restricted to one function only as was previously believed. To give an example, it is now known that Nox2 is not only expressed in macrophages and neutrophils as a defense enzyme, but also in other somatic tissues like epithelial cells of the colon, where it likely fulfils a signaling function. Fig. 4 shows an overview of the predominant expression patterns, functions, chromosomal locations, and protein co-factors of the seven enzymes.

### Nox2

The first human Nox enzyme studied in detail was Nox2 (formerly called gp91). Leukocytes from patients carrying the X‑chromosomal recessive form of chronic granulomatous disease (CGD) were found to be defective in the production of superoxide and clinically presented with severe granuloma and early death due to multiple infections. Cloning of the gp91 gene was one of the first examples of positional cloning of a human gene by chromosome walking [12, 13]. The other six human Nox genes and proteins were identified later based on the emerging human genome sequence, cDNA cloning, and the generation of monoclonal antibodies. As in the case of Nox2, patient-derived cell cultures and the establishment of in vitro systems for activity measurements were essential. These additional NADPH oxidases are described below.

Research on human Nox2 started with clinical and genetic data on patients suffering from CGD and with biochemical measurements of the “respiratory burst” observed in macrophages and neutrophils isolated form these patients. In clarifying the role of Nox2 in the antibacterial defense activity of the innate immune defense, a collaboration within clinical medicine, the development of activity assays by biochemists, the discovery of the often short-lived ROS using biophysics methods, the development of in vitro systems derived from neutrophils and macrophages of patients suffering from the different clinical forms of CGD, and the genetic analysis of the families of these patients was necessary for progress to be made in this field. The history of this early exciting phase of Nox research is extremely well described in a recent review [14]. These investigations showed the role of Nox2 in the innate immune defense in conjuction with myeloperoxidase, NO synthase, and superoxide dismutase (SOD). The combined action of these macrophage enzymes results in the formation of highly aggressive small molecules. These include: (i) H_2_O_2_, (ii) through Fenton chemistry, OH radicals; (iii) peroxynitrite, (iv) hypochloric acid, and (v) chloramines [15]. Further degradative enzymes activated in the defense reaction are lysozyme and other enzymes capable of degrading bacterial cell walls.

The role of superoxide in cellular defense by macrophages was discovered by Babior [16] based on the discovery of superoxide as a biological molecule [17] and SOD [18]. An important finding leading to an understanding of the oxidative burst was the fact that inhibition of the mitochondrial respiratory chain with cyanide did not inhibit the respiratory burst, showing that it is independent of the respiratory chain. On the other hand, inhibiting glycolysis, and thereby also the formation of NADPH via the pentose phosphate pathway (PPP), stopped it [19]. In vitro experiments showed that the reaction (Fig. 1) is specific for NADPH, excluding NADH as a substrate.

The defense reactions take place in phagosomes, which are specialized endocytotic vesicles enclosing particles (like bacteria) that are foreign to the cells and, in the case of bacteria, are recognized via receptors for bacterial-specific surface structures [20]. The phagosomes, which may be much larger than ordinary endosomes, subsequently fuse with lysosomes and form the endo-lysosome. At this stage, the defense reactions are triggered mostly by assembly of the Nox2 holo-enzyme at the endolysosome membrane. Components of signal transduction such as nuclear factor (NF)-κB, protein kinase C, and mitogen-activated protein (MAP) kinases are involved in the activation reaction [10, 14]. The necessary components of the holo-enzyme are shown in Fig. 4. The chemically highly reactive small molecules mentioned above remain sequestered in this compartment (which is topologically outside the cell), thereby protecting the rest of the cell. Much less is known about the absolutely necessary down-regulation of this process. The ROS and RNS needs to be removed by the antioxidative machinery of the cell [21], the cellular debris needs to be exocytosed, amino acids and other small biochemical building blocks are recycled into cellular metabolism, and last but not least, proteins of the invader (in the form of their peptides) are exocytosed via the major histocompatibility complex (MHC) and presented to T‑cells. The role in adaptive immunity is not discussed here, as it lies outside the scope of the present review. As part of the whole process, the macrophage survives and is ready for a second round of activity [22].

Activity of Nox2 in the phagosome is triggered only after recruitment of a number of regulatory proteins to the phagosome membrane core component gp91 (a highly glycosylated protein), among them p22 (intrinsic membrane protein) and the cytoplasmic regulatory subunits p47, p67, p40, and rac [10, 14]. A key step in this activation process is phosphorylation of PKCδ in its regulatory loop and, subsequently, phosphorylation of the Nox2 regulatory subunit p47 at the C‑terminal serine-rich autoinhibitory domain [23]. Again, genetic and biochemical analysis of these regulatory components was only possible through a combination of several complementary techniques of molecular biology, including clinical work with patients and patient cells. An array of milder forms of CGD along with other chronic inflammatory diseases caused by the loss of the regulatory subunits of Nox2 has been studied.

The main facts that were learned through the clinical phenotypes of these patients are the following:

I. The innate defense system of Nox2 is necessary for survival. The X‑chromosomally inherited recessive defect of gp91 (the catalytic subunit of Nox2) leads to death in early childhood by recurrent and severe infections, a condition known as chronic granulomatous disease (CGD).
II. Defects in the regulatory subunits normally lead to less severe phenotypes, depending on residual enzyme activity, and patients survive for longer. These clinical forms of CGD are inherited in an autosomal recessive manner and have been found in the genes coding for p22 (chr. 16), p40 (chr. 22), p47 (chr. 7), p67 (chr. 1), and rac (three different isoenzymes, not directly binding to Nox2) [14].
III. Membrane preparations and cytoplasmic fractions from the patients could be reconstituted to produce an in vitro cell-free system producing superoxide. In these reconstituted systems, gp91 and p22 are found to be localized in the membrane fractions, while the other four subunits (p40, p47, p67, and rac) are found in the cytoplasm. Antibodies were generated against all subunits, the proteins were characterized, and the genes cloned by cDNA cloning. Specific clinical variants of CGD were lacking the regulatory subunits.
IV. A defect of the innate immune system led to a defect in the adaptive immunity, affecting for instance long term B‑cell memory, presumably due to a defect in ROS production [24], pointing to close interaction between innate and adaptive immunity. The role of Nox2 in adaptive immunity (B-cell memory) is presently based only on a clinical correlation found in CGD patients.

The regulatory subunit of the Nox2 multisubunit complex, p47 (discussed above), has also been recognized as a general regulator of inflammatory diseases and was named neutrophil cytoplasmic regulatory factor (Ncf1) [25]. Surprisingly, the H_2_O_2_ that is produced (indirectly) by the Nox2 system of neutrophils is also a regulator of chronic inflammation, and a lack of ROS production by this system leads to autoimmune inflammatory diseases such as arthritis and a number of additional chronic inflammatory diseases (inflammatory bowel syndrome, lupus erythematosus). Patients presenting with CGD frequently suffer from inflammatory bowel syndrome. The mechanistic link between Nox2 and these unexpected associated diseases has been studied in detail. No less than three of the regulatory subunits of Nox2, as well as gp91 itself, have been found to be associated with different well-known chronic inflammatory diseases: a defect of gp91phox is associated with systemic lupus erythematosus (SLE) in human patients and genetically modified mice [26]; p47 (Ncf1) is correlated with chronic arthritis (reviewed in [25]); p67 (Ncf2) is correlated with SLE [27]; and p40 (NCF4) is correlated with atopic dermatitis [28] and inflammatory bowel disease [29]. Formal evidence of a direct causal relationship, however, is difficult to provide in these cases. The conclusion from these surprising findings is that Nox2 in human neutrophils and macrophages has two relatively different functions: for a limited amount of time in acute infections, the system produces the respiratory burst that is needed as an essential part of innate immunity. But, at a much lower level of activity, chronic production of ROS (presumably the relevant molecular species is H_2_O_2_) is necessary as a signal to regulate autoimmunity and inflammation, and a defect in the system leads to a variety of well-known autoimmune inflammatory diseases.

One of the questions that are still partly open is the tight regulation in time and space of the respiratory burst, which seems to be necessary to avoid an overwhelming amount of ROS triggered by infection.

In resting cells, which can express the Nox2 subunits (neutrophils, monocytes, macrophages), a deleterious activation of the respiratory burst is prevented by the spatial separation of the membrane components from the cytoplasmic regulatory components [30]. The process of activation is triggered by the binding of trigger substances to surface receptors of the cells [31]; these can be formyl-methionyl-leucyl-phenylalanine (fMLF), a peptide that is prokaryotic-specific and indicates bacterial infection, or by the binding of particles such as whole bacteria (see above, [20]) or particles covered with immunoglobulin G (IgG).

The pathways originating from this point are different in neutrophils and macrophages [32]. In both cases, the dead material is further degraded in the lysosome of the cell and eventually recycled in the cellular metabolism; however, only macrophages can survive this process, while neutrophils die through apoptosis or NETosis [33].

The process involving the oxidative burst is not only restricted to the phagosome, it is also quickly down-regulated if further stimulating signals (further infectious agents) are no longer present. This is accomplished by two mechanisms: (i) the antioxidative machinery of the cell, including NADPH-dependent antioxidative enzymes that are highly conserved in all eukaryotes (discussed in detail in [21]) can remove the excess ROS, and (ii) the rapid degradation of the Nox2 enzyme complex through endoplasmic reticulum associated degradation (ERAD) in a recently discovered mechanism including the protein negative regulator of ROS (NRROS) [34].

To summarize what has been said thus far, one can state: (i) Nox2, the best known defense enzyme of the Nox protein family, not only has a highly regulated role as a massive producer of ROS, but it is also a signaling module, as shown by the phenotype of CGD and other chronic autoimmune diseases (discussed above); (ii) the function of Nox2 in the innate immediate immune response is closely interdependent with the adaptive immune system; (iii) the Nox2 gp91 system is predominantly expressed in phagocytic white blood cells like macrophages and neutrophils, but has also been detected in more recent times in other somatic cells to a minor degree [14].

### The other Nox and Duox enzymes of human cells

A brief overview of these six isoenzmes will be given. They all have either signaling functions or redox functions for specialized biochemical reactions. Table 1 shows an overview of these additional Nox enzymes.

**Table 1:** Overview of the seven human NADPH oxidases

	Expression high	Expression low	Biochemical function	Regulatory factors	Chromosomal localization
NOX1	Colon	Extra-embryonic endoderm	Signaling (e.g. via Ha-ras)	p22, NOXO1, NOXA1	Xq22.1
NOX2	Phagocytes	Colon neurons	Defense; immune regulation in colon and other tissues	p22, p47, p67, p40, rac	Xp21.1–p11.4
NOX3	Inner ear	Embryonic kidney, spermatogonial stem cells	Synthesis of otolith, biochemical details unknown	p22, NOXO1, NOXA1	6q25.3
NOX4	Kidney, blood vessels	Oligodendrocytes, PSC maintenance	Cell division signaling, biochemical details unknown; location: nucleus, endoplasmic reticulum, mitochondria	p22	11q14.3
NOX5	Lymphoid tissue, testis	B and T cells	Signaling, biochemical details unknown	Ca^++^	15q23
DUOX1	Thyroid apical plasma membrane	Airway epithelial cells	Peroxidase domain; function in thyroid not known	DUOXA1, DUOXA2, Ca^++^	15q21.1
DUOX2	Thyroid apical plasma membrane	Salivary gland, airways, prostate	Synthesis of thyroxine, biochemical mechanism not known in detail; peroxidase domain, cell cycle regulation	DUOXA1, DUOXA2, Ca^++^	15q21.1

For details of the incompletely known biochemical functions of the enzymes please see main text. The relevant references for the data given in the figure are to be found in the paragraphs headed Nox1–DUOX2

Nox1: This NADPH oxidase isoform was the first non-phagocytic isoform to be discovered displaying 58% amino acid sequence identity to the phagocyte enzyme [35–37]. Activity depends on p22 and on the p47 and p67 homologs, NOXO1 and NOXA1, respectively [38, 39]. It is located on the X chromosome of the human and mouse genome [40] and is preferentially expressed in human colon epithelial cells [38]. Although a number of splice variants have been described [41], they were not functionally analyzed.

The majority of papers on the physiological or pathophysiological function of Nox1 was published in recent years. Oncogene-induced senescence (OIS) in cells expressing *Ha-ras*^*val12*^ is caused by the activation of Nox1 [42]. Nox1 activation is accomplished by phosphorylation at threonine 429 by protein kinase C beta1, facilitating association with the NoxA1 regulatory subunit [43]. In the HepG2 tumor cell line, Nox1 activity seems to be responsible for metabolic restructuring (concerning for instance the Warburg effect), and Nox1 is therefore considered to be pro-tumorigenic [44]. Nox1 is strongly expressed in colon cells and seems to be required for wound healing after inflammatory disease of the colon [45, 46]. Surprisingly, the antioxidative enzyme peroxiredoxin 6 (Prdx6) is a required co-factor for Nox1 activity in wound repair in an animal model of colitis [46]. Loss of Nox1 can cause inflammatory bowel disease [47], as can certain defects in Nox2 expression (discussed above). Regulatory subunits of Nox1 in colon cells (NOXO1, NOXA1) have been identified and are distinct from the regulatory subunits of Nox2 [39]. Through interaction with ADAM17, Nox1 is reponsible for the growth and metastasis of certain forms of colon cancer [48].

Nox1 and Nox4 are needed for the formation of the extraembryonic endoderm [49].

Nox3: Nox3 was discovered together with two other non-phagocytic Nox-encoding genes and proteins (Nox4 and Nox5) [50]. The gene coding for Nox3 is located on human chromosome 6. Both the clinical and mouse reverse genetics data showed that a major function of this gene lies in the vestibular and cochlear part of the inner ear of mammals [51]. Genome wide association studies [52] confirm the Nox3 encoding locus as a major genetic factor associated with hearing loss. A loss of function of this gene leads to hearing loss and to a defect in balance. It is highly probable that the biochemical function of the superoxide produced is synthesis of the otolith. However, no detailed biochemical studies on this reaction have been published. The expression pattern of Nox3, both at the mRNA and protein level, is very specific. Expression in the inner ear is at least 50-fold higher than expression in other tissues (fetal organs like embryonic kidney and spermatogonial stem cells) [50, 53]. It appears that the membrane-bound subunit, p22, binds in vivo to Nox3 [54]. However, CGD patients that suffer from a defect of p22 do not present with hearing or balance problems, an observation which speaks against a necessary function of p22 for Nox3 activation. On the other hand, the organizing cytoplasmic subunit NOXO1 (homolog of p47) and the activating cytoplasmic subunit, NOXA1 (homolog of p67) both seem to be necessary for Nox3 function [10, 55]. A deletion of NOXO1 in the mouse mimics the phenotype of the Nox3 defect.

Nox3 isoforms due to differential splicing have not been studied in detail as yet. The activity of Nox3 in the inner ear seems to be constitutive, but it is not understood why continuous activity in this organ is necessary. An interesting new finding [56] is the activity of Nox3, together with Nox5 in the precursor cells of oligodendrocytes that form the myelin sheath of neurons in the brain. This finding is based on a cell line, MO3-13, which is similar to oligodendrocyte precursor cells. In those cells, Nox3 and Nox5 activity seems to be necessary for the differentiation of the precursor cells. However, these interesting findings have not been confirmed as yet by in vivo data.

Nox4 was discovered [57, 58] as an NADPH oxidase highly expressed in kidney, but it is expressed at a low level in a large variety of human tissues, including cancers, cancer cell lines, and embryonic tissues. It is encoded on human chromosome 11. Together with Nox2, Nox4 activity is necessary for the maintenance of stemness of induced pluripotent stem cells [59]. An excellent review on this human Nox isoform was published relatively recently [60]. The enzyme is peculiar and differs from all other human Nox enzymes in several ways: (i) it is the only human Nox enzyme that can produce H_2_O_2_ relatively directly [61]. This activity can be prohibited by exchanging a single histidine residue in the extracytoplasmic E‑loop of the Nox4 sequence [61]. The enzyme does not contain a peroxidase domain. (ii) Its activity depends on the membrane bound protein co-factor, p22, but not at all on the soluble co-factors, p67, p47, NOXO1, or NOXA1. (iii) However, unusual Nox activating factors have been identified that interact with the Nox4 or p22-Nox4 complex, like protein disulfide isomerase, RNA polymerase delta interacting protein 2, and others [62]. The enzyme is therefore constitutively active and perhaps regulated primarily through its subcellular location. Indeed, careful localization studies have been performed and the four different splice isoforms mentioned below are localized in different compartments of the cell. It may be too early to draw definite conclusions, but the splice isoforms may be dominant negative forms regulating Nox4 activity, and in one case gene regulation via interaction with transcription factors and chromatin modifying enzymes may be the physiological purpose of Nox4D, which is localized in the cell nucleus [63, 64] and certainly is an isoform inactive as an NADPH oxidase. Localization of the active form of Nox4 has been found in mitochondria [65], but also in the ER [62, 66], in focal adhesions [67], and the plasma membrane [61] depending on cell type and physiological (stressful) situation. The Nox4 locus is localized on chromosome 11q14.2–q21, the exonic structure (25 exons) is dissimilar to that of Nox1, 2, and 3, and the existence and significance of splice isoforms was studied [63, 68]. Nox4 expression seems to play a major role in all phases of cancer development (initiation, growth, epithelial to mesenchymal transition, cell migration, metastasis) [60, 69] and even in oncogene-induced senescence triggered by the oncogenic version of the *Ha-ras* proto-oncogene [70]. It is therefore an important aim of cancer research to develop specific inhibitors of Nox4, which has not been achieved up to now, despite testing of a large number of candidate compounds [60].

Nox4 is the closest human homolog of the only NADPH oxidase of *S. cerevisiae*, Yno1, discussed in the concluding part of this review.

Nox5 (discussed in detail in [71]) is the only human Nox isoform that is active independent of co-factor proteins. It is also the only mammalian Nox isoform that is absent from a whole group of mammalian species (rodents, tested in rats and mice). In humans, the protein is encoded in chromosome 15, position q23 [50, 72]. Six different and presumably active splice isoforms have been detected, and an unusually high number [10] of polymorphic alleles is found in the human population, which are all above the 1% level and are correlated with different ethnic groups. Some of these alleles are non-functional, but they are never found as homozygous loss of function forms, possibly indicating that some Nox5 activity is necessary for life. The protein is located in the plasma membrane and contains 4 EF-hand sequences to bind 4 Ca^++^ ions in its *N*-terminal cytoplasmic tail. Calcium ion binding as well as calmodulin binding to a C-terminal binding sequence together with serine/threonine-specific phosphorylation on Thr^494^ and Ser^498^ through protein kinase C is a necessary activation mechanism for this enzyme [73, 74]. Interestingly, a similar C‑terminal calmodulin-binding domain in Nox4 was found to be non-functional [73]. The function or expression pattern of Nox5, which has been studied most intensively, is the activity of this enzyme during the course of mammalian sperm maturation (excluding, of course, rodents) with a peak in pachytene [72]. Although the Nox5 mRNA shows a peak in pachytene that is no longer present in later stages of sperm maturation, the enzyme seems to be present in mature sperm and necessary for capacitation [75, 76].

However, the enzyme is also highly expressed in the germinal centers of lymph nodes and spleen, possibly in maturing B and T cells [72, 74], but not in circulating mature B and T cells. It has been found in various other fetal and adult tissues as well as in cancer cell lines [74].

Duox1 and Duox2: These two Nox homologs of humans (and of mammals in general) are both located predominantly in the apical plasma membrane of thyroid gland epithelial cells and share similar structural properties; however, they are functionally diverse. Both enzymes are *N*-glycosylated, stored in the ER, and transported to their final location via the Golgi apparatus with the help of two maturation factors, DUOXA1 and DUOXA2 [77]. They bind Ca^++^ ions in their EF-hand domains, contain a seventh transmembrane helix connected to an extracellular *N*-terminal peroxidase domain, and do not appear to require cytoplasmic activating subunits. The requirement for the membrane-bound p22 subunit is controversial in the literature [10]. As a separate thyroid peroxidase is needed for the synthesis of thyroxine by Duox2, the functionality and requirement of the peroxidase domain of DUOX2 is also somewhat controversial, as judged from congenital forms of thyroid deficiency [78, 79]. The two enzymes are encoded side by side on human chromosome 15 and are divergently transcribed from structurally different promoters. Duox2 is expressed in the thyroid cells’ apical membrane, in salivary glands, in airway epithelia, and in the prostate. Its physiological function clearly involves thyroxine synthesis, although the mechanistic details of the iodination reaction are unknown. A second function of Duox2 has been found more recently in a host defense reaction in airway epithelia and in epithelia of the gastrointestinal tract [80] and, together with Nox4, in cell cycle regulation of dermal fibroblasts through the p53 checkpoint system [81].

Duox1 is likewise expressed in the thyroid, airway epithelia, and prostate. The function of Duox1 in the thyroid gland is unknown. However, two very interesting new findings point to a possible role of this enzyme. A detailed study of the role of this enzyme in signal transduction in relation to epidermal growth factor receptor (EGFR) revealed that the target of ROS signaling is not only the PTP (discussed above), but also the non-receptor tyrosine kinase, src, and the EGFR tyrosine kinase through sulfenylation of Cys^797^ by H_2_O_2_ [82]. Both DUOX1 and Nox2 were responsible for this process in airway epithelial cells. Another highly interesting pathophysiological role for DUOX1 was revealed in the generation of ionizing radiation (IR)-induced thyroid cell genomic instability and cancer induction [83]. Thyroid glands and thyroid cells in vitro maintain a high DUOX1 activity for many days after IR exposure controlled by interleukin(IL)-13 and mitogen-activated protein kinase (MAPK) p38, which leads to H_2_O_2_ production causing DNA strand breaks, chromosome translocation, and cancer development. Of course, the connection of this interesting phenomenon to the physiological role of DUOX1 in the thyroid is unknown at present.

### Discovery of the yeast NADPH oxidase, Yno1

**Fig. 5:**
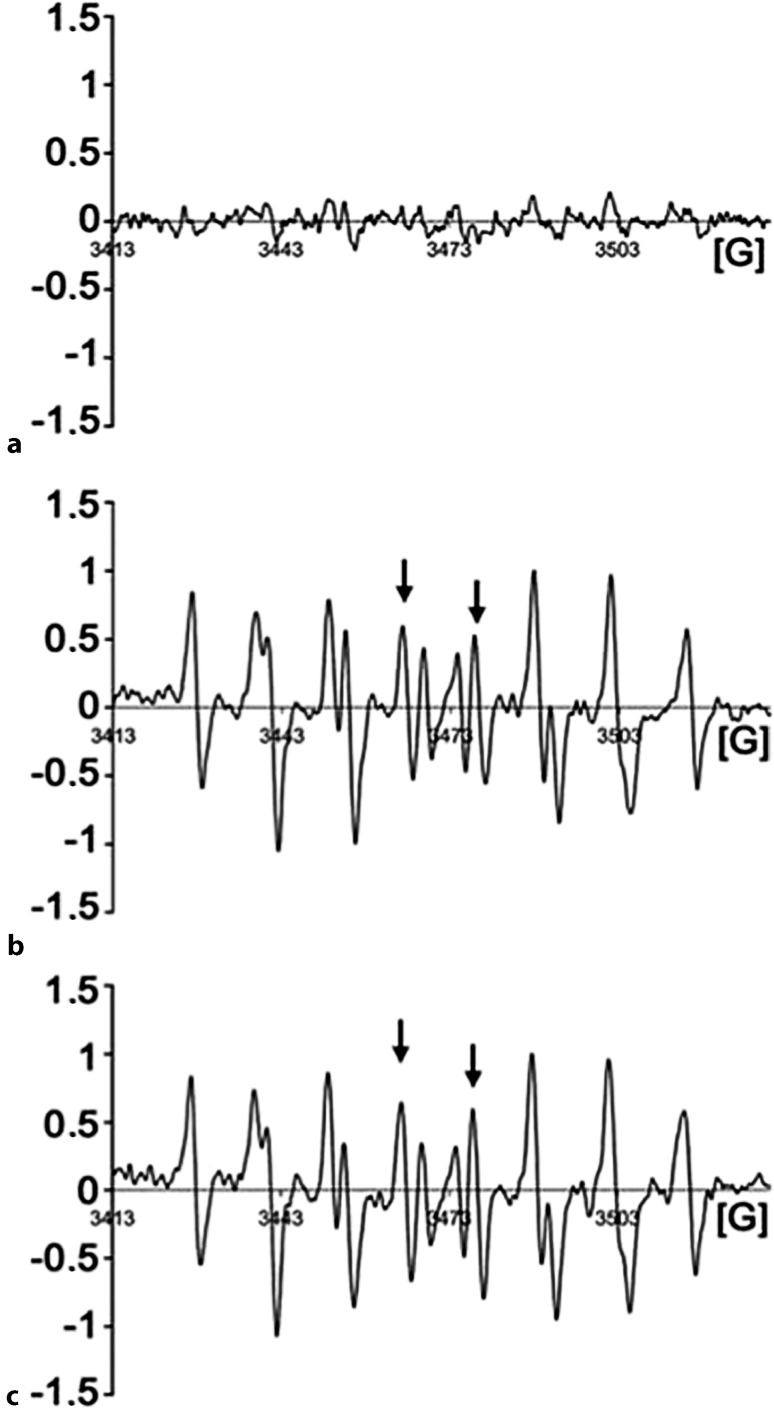
Electron spin resonance spectroscopy (ESR) spectra of *RAS2*^*gly18val19*^ yeast cells*. *The yeast “oncogenic” mutation *RAS2*^*gly18, val19*^ produces high levels of superoxide even in the absence of the mitochondrial respiratory chain. Therefore, the authors argued that a different source of superoxide must be present and tested all yeast genes for NADPH oxidase activity that show clear homology with the human NADPH oxidases. The figure shows in vivo measurements of the superoxide adducts of the spin trap DEPMPO by electron paramagnetic resonance (EPR). **a** 5-Diethylphosphono-5-methyl-1-pyrroline N‑oxide (DEPMPO) incubated with *RAS2* wild-type cells; no ESR signal is detectable. **b** DEPMPO incubated with *RAS2*^*ala18,val19*^ cells; the EPR spectrum clearly shows the presence of the DEPMPO superoxide adduct. **c** DEPMPO incubated with *RAS2*^*ala18,val19*^ respiratory deficient rho-zero cells. One sees the same amount of DEPMPO superoxide adduct as in **b**. (Modified from [84])

**Fig. 6:**
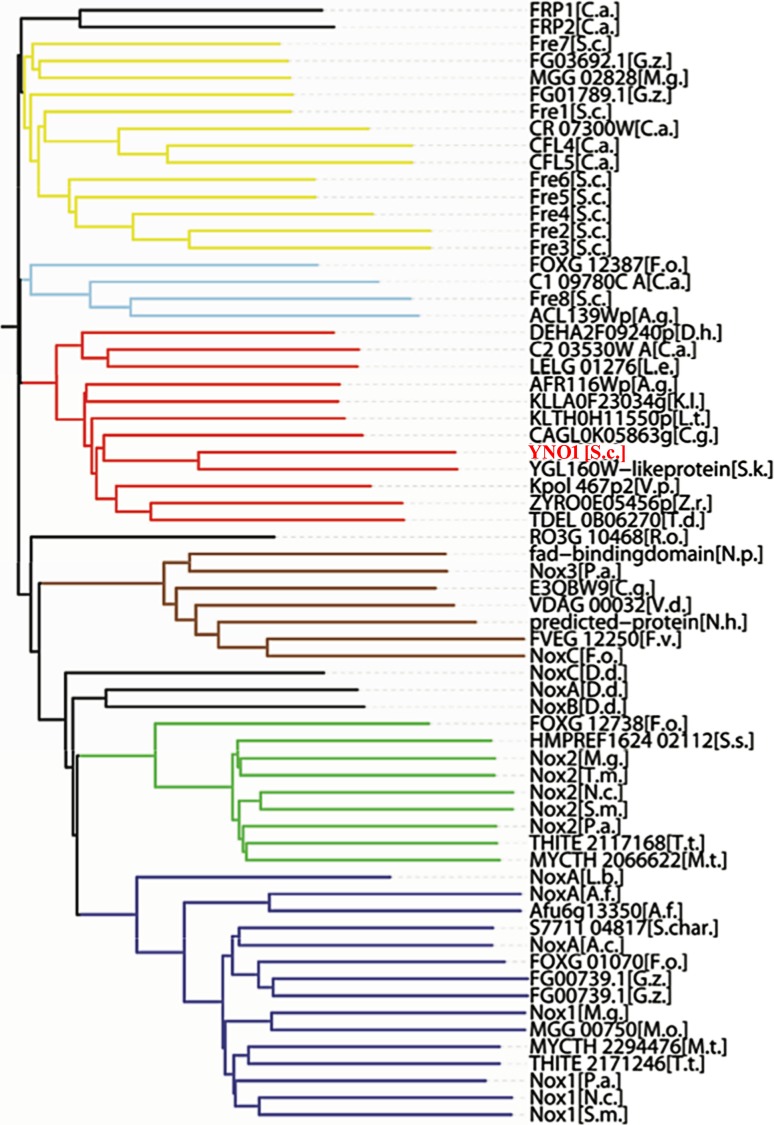
Phylogenetic relationships among the fungal members of the integral membrane reductase (IMR) protein superfamily. Only the gene names used in the sequence databases are used in the figure. Explanations of the abbreviations of the genus and species names are shown. The key result of this bioinformatic investigation is that the orthologs of Yno1 form a well–defined protein subfamily in the fungal kingdom, which is different from the previously known NoxA, B, and C subfamilies. The abbreviated species names in the cladogram are in alphabetical order:* A. g.* *Ashbya gossypii, A. c.* *Acremonium chrysogenum, A. f.* *Aspergillus fumigatus, C. a.* *Candida albicans, C. g.* *Candida glabrata, D. d.* *Dictyostelium discoideum, D.* *h.* *Debaryomyces hansenii, F. o.* *Fusarium oxysporum, F. v.* *Fusarium verticillioides, G. z.* *Gibberella zeae, K. l.* *Kluyveromyces lactis, L. e.* *Lodderomyces elongisporus, L. b.* *Laccaria bicolor, L. t.* *Lachancea thermotolerans, M. g.* *Magnaporthe grisea, M. o.* *Magnaporthe oryzae, M. t.* *Myceliophthora ahliaele, N. c.* *Neurospora crassa, N. h.* *Nectria haematococca, N. p.* *Neofusicoccum parvum, P.* *a.* *Podospora ahliae, R. o.* *Rhizopus oryzae, S.* *c.* *Saccharomyces cervisiae, S. char.* *Stachybotrys chartarum, S. k.* *Saccharomyces kudriavzevii, S. m.* *Sordaria macrospora, S. s.* *Sporothrix schenckii, T.d.* *Torulaspora delbrueckii, T. m.* *Togninia minima, T. t.* *Thielavia terrestris, V. d.* *Verticillium ahlia, V.p.* *Vanderwaltozyma polyspora, Z. r.* *Zygosaccharomyces rouxii*. (Modified from [86])

During the authors’ studies of yeast aging and apoptosis, a puzzling phenomenon was discovered: the well-known “oncogenic” point mutation, *RAS2*^*ala18, val19*^ led not only to unregulated growth signaling, lack of reserve cabohydrates, and a very short chronological lifespan (i. e., a defect of stationary phase survival), but also to death through apoptosis concomitant with a high level of superoxide production as discovered by dihydro ethidium (DHE) and electron paramagnetic resonance (EPR) spectroscopy monitoring [84, 85]. This observation was in perfect agreement with the discovery of oncogene-induced senescence in human cells expressing the homologous human *Ha-ras*^*val12*^ oncogenic point mutation [86]. More surprisingly, when the yeast cells expressing the dominant oncogenic version of *RAS2* were made rho-zero and could no longer produce superoxide in the respiratory chain, the ROS signal observed in these cells was unchanged and equally as high as in the respiring rho-plus cells, while the control cells (*RAS2* wild type) showed no such signal (Fig. 5; [85]). Seeing this, the authors decided to overexpress under galactose control all yeast genes that showed a marked sequence similarity to the catalytic subunit of human Nox2 and to monitor superoxide production during growth on galactose. The result was that only one of the nine open reading frames (ORFs) studied showed a strong production of superoxide (monitored by DHE fluorescence measurements and EPR spectroscopy). The authors named this gene *YNO1* (for yeast NADPH oxidase 1) and studied it in detail in vivo and in vitro [8]. The enzyme is located in the ER in growing and stationary yeast cells on glucose or on non-fermentable carbon sources. Purified microsomes from these cells and also purification of the enzyme and insertion into liposomes enabled in vitro studies showing specificity for NADPH with very little activity when NADH was used as an electron donor. The sequence of *YNO1* clearly showed that all known signature sequence elements of Nox enzymes were present (Fig. 3) and that a phylogenetic tree based on the neighbor joining method (Fig. 6) identified *YNO1* as member of a fungal subfamily of IMR proteins that showed little relationship with the previously known fungal Nox subfamilies, NoxA, B, and C (see legend to Fig. 6).

All other previously known fungal Nox proteins were found to be involved in multicelllular functions such as: plant pathogenicity [87] and cell differentiation like formation of the female sex organs and ascospore germination [88]. However, *S. cerevisiae* is a monocellular yeast and therefore the previously held notion of “NADPH oxidase: an enzyme for multicellularity” [89] is incorrect.

The physiological function of *YNO1* was studied on the one hand in situations where the yeast cells commit suicide, a process that is under genetic control and could even be advantageous for the yeast strain [90] or in situations where a profound metabolic restructuring is required, for instance during the diauxic shift when glucose is near exhaustion and the cells need to adapt to other respiratory carbon sources. Two papers appeared subsequent to the identification of *YNO1* as a Nox enzyme and shall be briefly summarized here. Leadsham et al. [91] showed that Yno1 in wild type cells is rapidly degraded by ERAD, but in respiratory-deficient *cox4* deletion strains this degradation is blocked, the cells express a high level of ROS originating from Yno1 and are killed by yeast apoptosis. Conversely, if also the *RAS2* gene is deleted in addition, Yno1 is degraded and the cells survive. These complex and interesting findings, among other things, shed light on the function of the yeast *RAS2* gene. Reddi et al. [92] showed that the yeast casein kinase γ orthologs, *YCK1* and *YCK2*, are regulated by Yno1 in conjuction with Sod1 (the cytosplasmic Cu/Zn superoxide dismutase). Yno1 is closely coordinated with Sod1 at the ER, where the yeast casein kinases are also located. The efficient, local, and transitory production of H_2_O_2_ stabilizes Yck1 and Yck2, preventing their destruction through the C‑terminal degron. This mechanism leads to respiratory repression and aerobic glycolysis, similar to the mammalian Warburg effect.

On the other hand, a number of published and unpublished results gained in the authors’ laboratory in close collaboration with the laboratory of Paul Cullen (Buffalo, N.Y.) point to the connection between Yno1 activity and the restructuring of the yeast cell’s actin cytoskeleton.

**Fig. 7:**
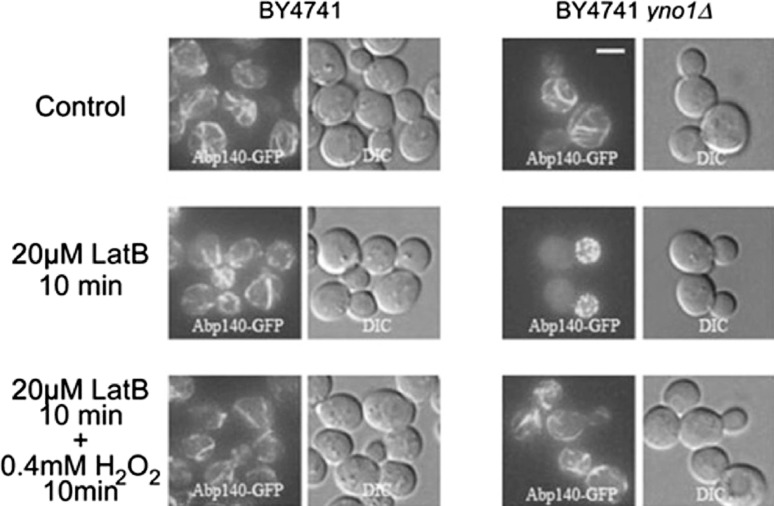
The role of the yeast Nox enzyme, Yno1, in actin cytoskeleton restructuring. F‑Actin was visualized with Abp140-GFP. Deletion of *YNO1* caused hypersensitivity to latrunculin B (inhibitor of F‑actin cable elongation) at a concentration of 20 μM, which does not inhibit F‑actin cable elongation in the WT. H_2_O_2_ reversed inhibition of actin cables by latrunculin B. Thus, reactive oxygen species produced by Yno1p are required for F‑actin regulation

Some evidence points to the fact that Yno1 is an enzyme involved in regulation of the actin cytoskeleton of yeast cells under conditions of stress where a restructuring of the actin cytoskeleton is necessary and that the relevant product of Yno1 is H_2_O_2_. The deletion of Yno1 is supersensitive to the drugs wiskostatin and latrunculin B and a low non-toxic concentration of H_2_O_2_ added in the culture media can restore growth under these conditions ([8]; Fig. 7). Latrunculin B at a concentration inhibiting growth leads to a complete loss of acin cables, while the F‑actin dots in the daughter cells are still visible. The addition of 0.4 mM H_2_O_2_ for only 10 min reverses this phenotype (Fig. 7). Restructuring of the actin cytoskeleton is a process needed for endocytosis and for the formation of pseudohyphae in strains derived from the Σ1278b strain that is closer to the *S. cerevisiae* wild type than the usual laboratory strains like BY4741. Deleting *YNO1* in such a strain leads to a marked defect in invasive growth and in pseudohyphae formation under special starvation conditions, which is the most common way of inducing pseudohyphae (unpublished results). The unpublished results and phenotypes described above are enhanced if the cells are made respiratory-deficient in addition to the *YNO1* deletion, pointing to the possibility that H_2_O_2_ production from *YNO1* and from the mitochondrial respiratory chain are synergistic for the signal transduction and actin-regulatory processes discovered.

### The role of human Nox4 in cancer biology

**Fig. 8:**
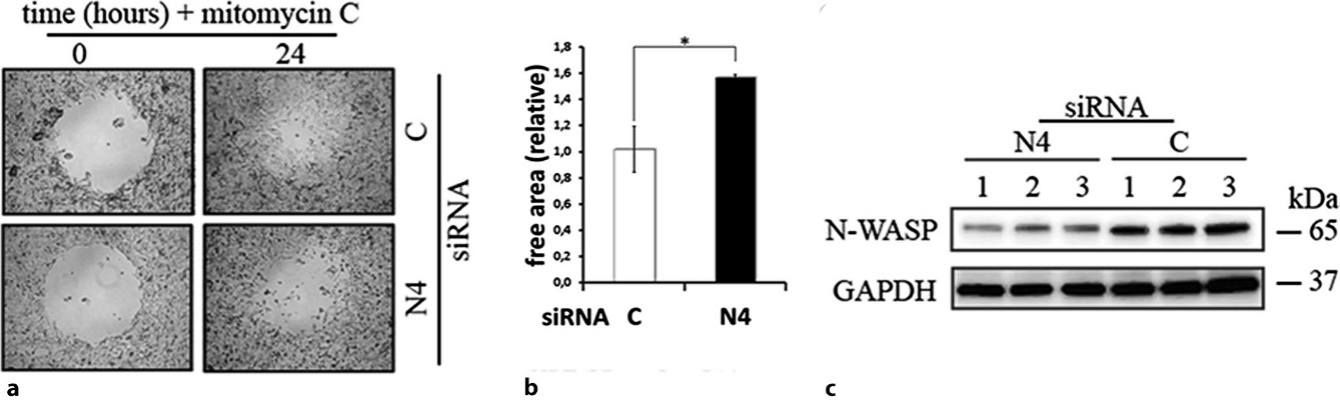
Cell migration assay performed with SH-SY5Y cells. **a** Cell migration assay using the SH-SY5Y neuroblastoma cell line. SH-SY5Y cells either transfected with Nox4 or scrambled small iRNA (*siRNA*) were grown to 80% confluency in a 24-well plate containing a hydrogel spot (time point 0 h). After removing the spot, the cells transfected with scrambled siRNA but not the Nox4 siRNA started to migrate into the cell free area (time point 24 h). **b** The remaining free area in the open spots was quantified. This shows that mobility of the neuroblastoma cells depends on Nox4 activtiy. **c** Under similar conditions, the actin cytoskeleton regulator neuronal Wiskott-Aldrich syndrome protein (*N-WASP*) is down-regulated as shown by Western blots normalized to the housekeeping protein glyceraldehyde-3-phosphate dehydrogenase (*GAPDH*) in the cancer cell line HepG2. *C* control (scrambled siRNA), *N4* Nox4 specific siRNA

Sequence comparisons of Yno1 with all seven human Nox enzymes show that Nox4 displays by far the largest similarity in sequence with Yno1 [93]. In both established tumor cell lines that the authors (HepG2 and SH-SY5Y), the enzyme, Nox4, was strongly expressed as compared with non-tumorous material from the same organ, and located in the ER, like the yeast enzyme. RNA interference (RNAi) experiments showed that transcriptional down-regulation of Nox4 led to the weakening or disappearance of actin stress fibers. This effect was again, like in the yeast cells, reversible by adding a low non-toxic H_2_O_2_ concentration. Scratch assays showed in the case of the SH-SY5Y cells that inhibiting Nox4, which leads to a loss of actin cytoskeleton restructuring, led to a loss of cell mobility (Fig. 8). As it is well known that cell mobility depends on the restructuring of the actin cytoskeleton and is essential for the process of metastasis, the authors propose here, like other authors [94], to study Nox4 as a drug target in cancer therapy. At present, there are no known pharmacological inhibitors that are specific for Nox4; however, such a substance would be highly desirable for a new concept of tumor therapy.

## Conclusion

NADPH oxidases currently play a prominent role in biological and medical science research. The present article reviews recent progress in this field with an emphasis on newly discovered facts about the physiological function of the seven human NADPH oxidases (Nox enzymes), in particular in signaling growth and cell differentiation through the second messenger, H_2_O_2_. A special role of the Nox system in regulation of the actin cytoskeleton was discovered through the work of the authors’ group and other groups. This is not only in relation to fungal model systems, in particular the pseudohyphal growth of *S. cerevisiae*, but also in relation to cell migration and possibly metastasis of human cancer cells.
